# A Clinician-Supported Mobile App to Reduce Mental Health Symptoms Among World Trade Center Responders in Florida: Protocol for a Randomized Controlled Trial

**DOI:** 10.2196/95229

**Published:** 2026-06-30

**Authors:** Mark J Macgowan, Kyle Possemato, Eric Kuhn, Nan Hu, Sandra Lowe, Stephanie Garcia, Isis Panellas, Arrianna Edwards, Victoria Gray, Ruth Marquez, Luciana Pitta, Roberto G Lucchini

**Affiliations:** 1 School of Social Work, Robert Stempel College of Public Health and Social Work, Florida International University, 11200 SW 8th Street, AHC5-513, Miami, FL, 33199, United States, 1 3053480427; 2 Syracuse VA Medical Center, New York, NY, United States; 3 VA National Center for PTSD, Palo Alto, CA, United States; 4 Department of Biostastistics, Robert Stempel College of Public Health and Social Work, Florida International University, Miami, FL, United States; 5 Icahn School of Medicine, Mount Sinai, New York, NY, United States; 6 Department of Environmental Health Sciences, Robert Stempel College of Public Health and Social Work, Florida International University, Miami, FL, 33199, United States, 1 3053480427

**Keywords:** World Trade Center, first responders, PTSD Coach, mobile health, randomized clinical trial, posttraumatic stress disorder, PTSD, depression, anxiety, sleep problems, mobile phone

## Abstract

**Background:**

World Trade Center (WTC) general responders (GRs) continue to experience long-term mental health conditions, including posttraumatic stress disorder (PTSD), depression, anxiety, and sleep disturbance. A growing number of GRs reside in Florida, where barriers such as stigma, limited access to specialty care, and age-related limitations contribute to persistent unmet mental health needs. PTSD Coach, a mobile app originally developed for trauma-exposed veterans, has shown promise but has not been evaluated with WTC GRs or adapted for Spanish-speaking responders.

**Objective:**

This study aims to evaluate the feasibility, acceptability, and efficacy of Clinician-Supported (CS) PTSD Coach for reducing PTSD symptoms among English-speaking and Spanish-speaking WTC GRs living in Florida. Secondary objectives include evaluating effects on depression, anxiety, and sleep disturbance; comparing CS PTSD Coach with Self-Managed (SM) PTSD Coach and a waitlist control; and adapting and delivering a Spanish-language version of the intervention.

**Methods:**

This study is a 3-arm randomized controlled trial comparing CS PTSD Coach, SM PTSD Coach, and a waitlist control for reducing PTSD, depression, anxiety, and sleep disturbance among English-speaking and Spanish-speaking WTC GRs living in Florida. A total of 120 participants are randomized and assessed at baseline, 8 weeks, and a 12-week follow-up. CS PTSD Coach includes 4 clinician-guided sessions integrated with app-based activities, whereas SM PTSD Coach includes a single orientation session. Outcomes include PTSD symptoms (PTSD Checklist for DSM-5), depression (Patient Health Questionnaire-9), anxiety (Generalized Anxiety Disorder-7), and sleep disturbance (Insomnia Severity Index). Feasibility and acceptability are assessed using app use data, satisfaction ratings, and usability measures. Primary analyses focus on between-group differences after the intervention (8 weeks), with secondary longitudinal analyses incorporating all assessment time points.

**Results:**

The notice of award was received in September 2024, and institutional review board approval, including amendments, was granted in January 2025. Recruitment began in February 2025, with data collection initiated in March 2025. As of April 2026, 63 participants have been enrolled, and outcome data collection is ongoing. Data analysis will commence following completion of follow-up assessments, with dissemination of results anticipated in 2027.

**Conclusions:**

This trial will provide the first randomized controlled evaluation of a clinician-supported mobile PTSD intervention for WTC responders, including Spanish-speaking GRs. Findings will inform the feasibility, acceptability, and potential scalability of supported digital mental health interventions for aging, geographically dispersed disaster-exposed populations.

## Introduction

### Background and Rationale

The World Trade Center Health Program (WTCHP), funded by the Centers for Disease Control and Prevention and the National Institute for Occupational Safety and Health (NIOSH) since 2011, provides medical monitoring and treatment for September 11, 2001 (9/11)-related conditions to more than 140,000 individuals, with enrollment increasing by about 6% annually [1,2]. Research on the World Trade Center (WTC) cohort has documented WTC-related mental health conditions that include posttraumatic stress disorder (PTSD) as the most commonly certified psychiatric condition with a prevalence ranging from 3.8% to 29.6% [3], as well as anxiety, depression, substance abuse, and sleep disturbances [4,5]. Most of these conditions show a chronic or persistent course, with symptoms lasting for years after exposure. As of September 2023, the WTCHP has certified WTC-related mental health conditions in about 17% of responders and survivors [1,2].

Many general responders (GRs)—workers or volunteers involved in rescue, recovery, cleanup efforts, or related support services—experience significant symptoms of subthreshold PTSD (ie, <30 on PTSD Checklist for DSM-5 [PCL-5]) [6,7]. Overall, 26.8% of police and 46% of nontraditional responders (eg, construction and demolition workers, and volunteers) met the criteria for probable WTC-related full or subthreshold PTSD an average of 12 years after 9/11 [8]. GRs with subthreshold PTSD commonly experience significant distress and long-lasting functional impairment related to specific sets of symptoms, such as hypervigilance and avoidance [9], which highlight the need for assessment and treatment [8]. Almost 20 years after 9/11, nearly half of nontraditional responders and a fifth of police responders reported a need for mental health support [10].

However, rates of those seeking mental health services are lower than expected [11], and perceived unmet mental healthcare need is persistent [12,13]. A focus group study on WTC GRs underlined the systematic challenges to accessing mental health care [14]. Some of the challenges included stigma, lack of institutional recognition of PTSD, limited treatment options, and practical obstacles such as transportation and age-related bias. These recurring challenges underscored the study’s conclusion that responders face entrenched, system-level obstacles to obtaining needed care.

In 2012 and 2014, the WTCHP Scientific Technical Advisory Committee recommended research on mental health interventions [15] and the *“*effectiveness and utility of PTSD treatments*”* [16]. A 2020 review based on the National Institute of Environmental Health Sciences translational research framework [17] noted that although clinical interventions in the WTCHP included a wide range of options [3,18-24], there were few controlled studies. Most interventions were delivered in person with highly exposed individuals [3]. At the time this study was developed, no studies were conducted with mobile app–based remote interventions to serve the large number of GRs with full or subthreshold PTSD. Furthermore, no interventions focused on Spanish-speaking Hispanic GRs have been conducted.

Studies of PTSD interventions for which current evidence has been supportive in other populations (eg, military veterans) can be used to advance care for GRs along the translational spectrum [17]. Furthermore, WTC researchers have strongly recommended trials that include tele-mental health [17].

### Growing WTC Subcohort in Florida

The number of eligible WTCHP members relocating to Florida has steadily risen over the years. National distribution figures of WTC members with a National Providers Network assignment show Florida as the largest location [25]. Additional data from the WTCHP General Responder Data Center (GRDC) reported that 7346 GRs were registered with a Florida address in 2025 [26]. In collaboration with the WTCHP GRDC, our research group conducted a secondary data analysis of the data on GRs living in Florida in 2023. The WTC GRs in Florida, for whom monitoring data were available (N=3124), were on average aged just over 60 years (61.4, SD 8.5 years), frequently retired (n=1252, 44.6%), male (n=2292, 81.6%), and of Hispanic ethnicity (n=727, 25.9%). Some WTC GRs (n=370, 15.1%) had a WTC-related mental health condition.

In addition to the secondary data analysis, our team surveyed Florida WTC GRs in August 2023 about their research interests. A total of 244 GRs responded and expressed strong interest in PTSD and sleep-related studies (n=127, 52% and n=185, 76%, respectively). Although the preference for receiving support was for in-person interventions (n=107, 44%), 22% (n=44) of respondents expressed a preference for app-based or remote modalities, and an additional 44% (n=107) reported no opposition to their use.

This study responds to one of the research areas of the Zadroga Act (Title 42 USC §300 mm–51) to treat *“*WTC-related health conditions for which there has been treatment uncertainty*”* and, by including Spanish-speaking Hispanic GRs, helps advance health equity as required in Notice of Funding Opportunity issued by the National Institutes of Health Office of Human Resources (NOT-OH-23‐002).

The focus on this subcohort of the GRs in Florida is especially relevant, given that members generally relocate to Florida upon retirement and are older compared to GRs living elsewhere. As this subcohort continues to grow, aging may intersect with the long-term mental health effects associated with 9/11 exposures. Relocation can disrupt continuity of mental health care; embedding a supported digital intervention within the WTCHP and the National Providers Network may help sustain engagement and reduce symptoms during relocation, including among Spanish-speaking responders.

Persistent symptom burden alongside underuse of care reflects enduring barriers, including stigma, travel demands, limited specialty availability, and challenges faced by retired or older responders, such as reduced mobility and progression of medical conditions. App-based, clinician-supported care offers a scalable approach to addressing these barriers while preserving trauma-informed treatment strategies in a geographically dispersed cohort.

### Aims of the Study

The overarching goal of the study is to assess the feasibility, acceptability, and efficacy of Clinician-Supported (CS) PTSD Coach, originally developed for English-speaking military veterans [27,28] but never tested with WTCHP members, for reducing symptoms of PTSD, depression, anxiety, and sleep disturbance among multiethnic English-speaking and Spanish-speaking GRs in Florida. The four primary aims are to (1) assess the feasibility of CS PTSD Coach with GRs living in Florida; (2) evaluate the acceptability of CS PTSD Coach; (3) determine the efficacy of CS PTSD Coach in reducing PTSD, depression, anxiety, and sleep symptoms through a controlled trial; and (4) develop and deliver a Spanish-language version of CS PTSD Coach.

### Hypotheses and Exploratory Analyses

If both the CS and SM PTSD Coach interventions demonstrate improvements relative to the waitlist control, we will conduct direct comparisons between CS and SM PTSD Coach to evaluate differential symptom reduction. We hypothesize that CS PTSD Coach will result in greater reductions in PTSD, depression, anxiety, and sleep disturbance due to the addition of clinician support.

We also explore potential moderators and mediators of treatment effects, including baseline PTSD severity, sex, race, ethnicity, language, and engagement level. These analyses are exploratory and intended to inform future hypothesis-driven studies.

The fourth aim focuses on developing and delivering a Spanish-language version of CS PTSD Coach. The existing English-language clinician manual has been adapted for 9/11 responders and translated into Spanish. The adapted intervention is delivered by bilingual or bicultural clinicians, and its feasibility, acceptability, and preliminary efficacy evaluated using the same measures as the English-language intervention.

## Methods

### Study Design

The study design in the 2-year project includes a 3-arm randomized controlled trial (RCT) of the experimental intervention CS PTSD Coach, compared to Self-Managed (SM) PTSD Coach and a waitlist control, with each group assessed at baseline, immediate postintervention (8 weeks), and follow-up (12 weeks). The RCT design combines feasibility, acceptability, and efficacy based on CS PTSD Coach effectiveness with English-speaking veterans in the Department of Veterans Affairs (VA) [27,28]. Feasibility, acceptability, and efficacy data will fill the gap of knowledge on mental health interventions specifically for the WTC GRs and especially for the Spanish-speaking population. This design achieves a balance between efficacy (internal validity) and clinical utility (external validity) [29] by including an RCT and collecting data on the acceptability and feasibility of both the CS and SM versions of PTSD Coach for English and Spanish speakers. Although we predict that the CS version will be more efficacious than SM PTSD Coach, given the added support [27,30], the design also yields valuable information about 2 interventions.

In addition to efficacy and clinical utility, additional strengths include manualized treatment and theory-driven and data-driven hypotheses and treatment. Additionally, we plan to explore several potential moderators and mediators, which may predict differential treatment outcomes.

The trial was registered with ClinicalTrials.gov (NCT06648928) before participant enrollment. Protocol development and reporting followed the SPIRIT (Standard Protocol Items: Recommendations for Interventional Trials) 2013 guidelines for clinical trial protocols.

### Randomization and Groups

**Figure 1.**
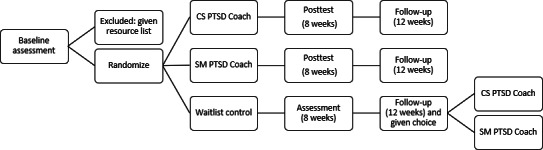
Study design. CS: Clinician-Supported; PTSD: posttraumatic stress disorder; SM: Self-Managed.

As indicated in Figure 1, after baseline assessment, participants are assigned based on a random number generator on a rolling basis into three groups: (1) experimental treatment CS PTSD Coach, (2) alternative treatment SM PTSD Coach, and (3) a waitlist control that receives no treatment until after the follow-up assessment. Allocation concealment is supported by using sequentially numbered, opaque, sealed envelopes prepared by an individual not involved in assessment or intervention; envelopes are opened sequentially after participant enrollment.

After assignment, participants receive CS PTSD Coach, SM PTSD Coach, or the waitlist control group. Participants in all 3 groups are assessed at 8 weeks (the typical duration of the CS PTSD Coach) and 4 weeks later at follow-up (12 weeks). At that point, participants in the waitlist control group are given the choice of CS or SM PTSD Coach but are not further evaluated. This approach was adopted for ethical reasons to provide all participants with a choice of treatment at the end.

### Participants

WTC GRs currently living in Florida, of any age, with PCL-5 scores consistent with subthreshold or clinical PTSD (eg, *>*27) are included. Participants are introduced to the project through a contact via SMS text messaging, email, or letter by the recruitment team. To be included, participants must have a smartphone capable of running PTSD Coach (available on both Android and iOS platforms) and be fluent in either English or Spanish.

Exclusion criteria determined by the initial screening with the assessors include (1) active or recent (past month) suicidal ideation or attempt; (2) severe alcohol use measured by an Alcohol Use Disorders Identification Test score of 20+ [31]; (3) except for cannabis, no use of illicit or unprescribed drugs in the last 2 weeks as indicated on the American Psychiatric Association Level 2 Substance Use Measure [32]; (4) presence of neurologic or neurodegenerative conditions associated with significant cognitive impairment measured using the Blessed Orientation-Memory-Concentration test [33]; and (5) current mental health counseling for PTSD (primary outcome) to avoid confounding effects. Participants endorsing active suicidal ideation during screening receive an immediate safety evaluation and are provided referrals to appropriate mental health services or emergency care, with follow-up outreach conducted when clinically appropriate.

### Recruitment

Recruitment benefits from the network established in an ongoing survey targeting Florida WTC GRs and is supported by bilingual outreach staff. The recruitment strategy uses two consecutive approaches: (1) direct contact via email, phone, and home address of WTCHP members who consented to be contacted for research, with information provided by the WTCHP GRDC and (2) public outreach through university and electronic releases, a website, and participation in local emergency responder events, facilitated through established contacts with WTC firefighters and law enforcement representatives residing in Florida.

### Sample Size Calculation

The rationale for selecting a sample size of 120 participants, 40 per group across the 3 arms of the RCT (CS PTSD Coach, SM PTSD Coach, and waitlist control), is based on achieving sufficient statistical power to detect meaningful differences in the outcomes. As the primary analysis is to compare the preintervention (baseline) and postintervention (8 weeks) change in outcomes across intervention arms, analysis of covariance (ANCOVA) serves as the primary between-group analytic approach as it is the most powerful model for pre- and postanalysis. Specifically, with 10% expected data attrition, this sample size will provide 80% power to detect a minimal difference of 0.30 SDs between-group means using ANCOVA with a significance level of *P*<.05. The design assumes a common within-group SD of 1.00 and covariates with an R-squared of 0.20, ensuring robust comparisons across interventions while accounting for potential confounding variables.

### Interventions

PTSD Coach was developed by the VA’s National Center for PTSD Dissemination and Training Division as a self-managed mobile app that contains 4 sections: learn, self-assessment, manage symptoms, and find support [34]. The app is available at no cost from the App Store and Google Play marketplaces [35] and thus reaches almost all (99.5%) US smartphone users [36]. For this research project, the app is available in both English and Spanish. The primary focus of PTSD Coach is on PTSD symptoms, which include (1) reexperiencing, such as having intrusive traumatic symptoms (eg, flashbacks and nightmares); (2) persistent avoidance of stimuli associated with the traumatic event; (3) negative thoughts or feelings that worsened after the traumatic event; and (4) hyperarousal, such as sleep disturbance, excessive startle reactions, and irritable behavior [37]. PTSD Coach provides psychoeducation on trauma and PTSD and helps trauma-exposed individuals to monitor and manage traumatic stress symptoms. PTSD often cooccurs with depression and anxiety, and PTSD Coach is also effective on these symptoms [38], but it was never studied with GRs. Sleep disturbance is a common complaint among WTC responders [5], and veterans have reported that the mobile app has helped them with sleep problems [34]. In summary, the PTSD Coach app focuses on targeting the primary mechanisms of PTSD and associated symptoms of depression, anxiety, and sleep disturbance.

CS PTSD Coach was developed as a brief intervention for veteran primary care patients with elevated PTSD symptoms who were unlikely to use a mobile app on their own [34,39]. CS PTSD Coach is a manualized intervention that combines the PTSD Coach mobile app with four 20-to-30-minute sessions delivered over 8 weeks. Sessions focus on instructions for app use, setting symptom reduction goals, and assigning PTSD Coach activities (ie, symptom monitoring, management strategies, and psychoeducational readings) for completion between sessions. The clinician sessions help participants select the management strategies within the app that can best address their PTSD symptoms and incorporate these strategies into their daily lives. As part of aim 4, the existing treatment manual developed for veterans will be adapted for the GRs, including translating it into Spanish. The main components of each session are shown in Table 1.

**Table 1.** Clinician-Supported PTSD Coach overview and fidelity elements.

	Session 1	Sessions 2 and 3	Session 4
Format	Audio or audio and video	Audio or audio and video	Audio or audio and video
Content	Overview of treatment Instructions for app use Discuss learn modules Complete PCL-5^a^ in app Target problem domain Try 1-2 manage strategies	Review homework Discuss change on PCL-5 graph Discuss manage strategies used Target new symptom domain to manage Problem solve any adherence issues	Review homework Discuss Learn, assessment, and manage tools used since last session Recommend treatment based on PCL-5 score Make treatment or self-management plan
Homework	Complete PCL-5 weekly Read at least 2 Learn topics Use manage strategies daily	Complete PCL-5 weekly Read at least 2 Learn topics Use manage strategies daily	Follow through on treatment plan Continue to use app as desired

a PCL-5: PTSD Checklist for DSM-5.

CS PTSD Coach is delivered by bilingual or bicultural master’s or doctoral students in social work with experience in providing remotely delivered psychosocial care, supervised by a Florida-licensed clinician. The university is a Hispanic-serving institution; most students are bilingual or bicultural Hispanics. The intervention developer leads clinician training that includes didactic training and role plays. During treatment delivery, clinicians participate in weekly group consultation calls with the intervention developer’s team. The clinicians complete fidelity checklists of essential protocol components following each session. An independent fidelity assessment will be conducted using audio-recorded sessions for 25% of sessions randomly selected from each of the 4 sessions of the protocol.

The SM PTSD intervention includes 1 brief session with the clinician who provides basic information about downloading and using the app [27]. There are no further instructions with the participants in this group.

### Measures

#### Overview

The project includes measures to assess clinical outcomes and exploratory implementation (process) outcomes, including satisfaction, feasibility, and acceptability. All outcome measures are administered by research assistant assessors who are blind to conditions and not involved in treatment delivery. The clinical measures are brief, have good psychometrics, and have been used in clinical trials, so they are sensitive to treatment change (when needed, Spanish versions of the measures will be used). Some of the instruments have been used in the WTCHP monitoring program, so would be familiar to respondents. To promote retention and completion of follow-up assessments, participants are contacted using multiple methods and reminded of upcoming assessments, and outcome data are collected remotely to minimize participant burden.

#### Clinical Outcomes

The primary clinical outcome variable, PTSD symptoms, is assessed at all 3 time points (baseline, postintervention, follow-up) using the PCL-5, which has good reliability and validity [7,40]. The PCL-5 is used to assess changes in treatment response, with items rated on a 0 (not at all) to 4 (extremely) scale and higher total scores reflecting greater PTSD symptom severity. Studies of expected minimal important difference with the PCL-5 indicated that changes under 5 are unreliable and 9 to 12 points reflect real change [41]. Thus, we expect at least a 5-point difference to be reliable change and 9 to reflect clinically meaningful differences. As few PTSD Coach studies have explored changes in specific PTSD symptom clusters [38], we will conduct exploratory subscale analyses to identify which clusters respond best to the intervention.

Depression severity, a secondary clinical outcome, is assessed at all 3 time points using the Patient Health Questionnaire-9 (PHQ-9), a reliable and valid measure [42,43] used in similar studies [28,44]. The PHQ-9 is a 9-item self-report measure of depression symptoms in the past 2 weeks. Participants rate each item on a 0 (not at all) to 3 (nearly every day) scale. A score of 5 indicates mild depression, 10 to 14 moderate, 15 to 19 moderately severe, and 20 to 27 severe [43]. Scores ranging from 8 to 11 suggest major depressive disorder [45].

Anxiety, a secondary clinical outcome, often cooccurs with PTSD [37] and is assessed at the 3 time points using the Generalized Anxiety Disorder-7 (GAD-7)[46], a 7-item self-report measure of general anxiety symptoms over the past 2 weeks. Respondents rate their anxiety on a scale from 0 (not at all) to 3 (nearly every day). The measure has good reliability and validity [46,47] and has been used with first responders [44]. A score of 8 or higher indicates probable generalized anxiety disorder [48,49], with cut points of 5, 10, and 15 indicating mild, moderate, and severe levels of anxiety, respectively [46].

Sleep disturbance symptoms, a secondary clinical outcome, is assessed using the 7-item self-report Insomnia Severity Index (ISI) [50,51]. The ISI assesses the severity and effects of insomnia symptoms over the past 2 weeks. Items are rated on a scale from 0 (no problem) to 4 (very severe problem), yielding a range of 0 to 28, with higher scores reflecting increased severity of insomnia symptoms. Scores of 0 to 7 indicate absence of insomnia, 8 to 14 subthreshold insomnia, 15 to 21 moderate insomnia, and 22 to 28 severe insomnia [50]. For clinical trials, a cut score of 11 is recommended [51], with a score of 15 indicating clinically significant insomnia symptoms [50]. A reduction of >7 points indicates optimal change for moderate improvement and a reduction of >8 points indicates optimal change for marked improvement [51].

#### Satisfaction, Feasibility, and Acceptability

In addition, several exploratory implementation (process) outcomes are intended to capture intervention uptake, user experience, and implementation potential rather than clinical efficacy. Satisfaction with treatment is assessed using the Client Satisfaction Questionnaire, a widely used 8-item self-report measure assessing patient satisfaction with care [52,53]. Each question is rated on a scale from 1 to 4, with higher scores reflecting increased satisfaction.

To assess feasibility, responder engagement with the app will be evaluated using objective use data from the app developer (VA Palo Alto Health Care System), including frequency and duration of use, peak use periods, and module-level engagement.

Treatment acceptability and appropriateness is assessed with the Acceptability of Intervention Measure and Intervention Appropriateness Measure, both consisting of 4 items with good reliability and validity [54]. Each item is rated on a scale from 1 (completely disagree) to 5 (completely agree), with higher scores indicating more acceptability and appropriateness.

To assess usability of CS PTSD Coach and the mobile app itself (depending on intervention group), we use the System Usability Scale [55] that consists of 10 items rating users’ perceived usability of a system, which has strong reliability and validity [55]. Higher scores indicate greater usability. We expect “good” usability, defined as a score of >68 [55].

### Data Collection and Management

Data are captured and managed using a secure Research Electronic Data Capture database (REDCap; Vanderbilt University) [56], created and maintained at the statistical consulting center of the host university, Florida International University. The database includes value, range, and logic checks, and undergoes 2 levels of internal system testing and revision to ensure data integrity. Data collection follows the schedule outlined earlier, which includes baseline sociodemographic information; mental health assessments (PTSD, depression, anxiety, and sleep disturbance); and postintervention evaluations of satisfaction, feasibility, and acceptability. University-based assessors enter data directly into REDCap, linked to unique IDs. Access to participant identifiers is restricted to the study coordinator and data entry team, and REDCap’s built-in quality checks help detect inappropriate values.

All data are securely stored in the Amazon Web Services cloud with encryption both in transit and at rest. The REDCap environment is protected by virtual firewalls and requires 2-factor authentication for access. App usage data collected by the VA will be identified solely by randomly generated invitation codes, ensuring full de-identification. The host university will maintain a secure linkage file connecting app use data with participant identifiers. Upon completion of data collection, deidentified data will be exported for analysis. Long-term data access and archival will follow the host university’s Division of Information Technology Security Office policies, ensuring compliance with health information privacy standards.

### Statistical Analysis Plan

In aim 1 (feasibility), we will assess the feasibility of PTSD Coach with multiethnic GRs in Florida. App use data obtained from the VA will be summarized descriptively with mean (SD) for continuous variables (eg, time spent in the app) and frequency (N) and percent (%) for categorical variables (eg, modules completed). The use patterns will be examined by the intervention group (CS vs SM) and language (English vs Spanish) used.

In aim 2 (acceptability), we will evaluate the acceptability of the CS PTSD Coach intervention. Satisfaction is assessed using the Client Satisfaction Questionnaire, with ratings of “good” or “excellent” indicating endorsement. Acceptability and appropriateness are measured using the Acceptability of Intervention Measure and the Intervention Appropriateness Measure, with agreement ratings serving as indicators of positive reception. Data will be summarized with mean (SD) for continuous variables and frequency and percent for categorical variables. The data will be summarized for the entire cohort and by both intervention and language groups.

In aim 3 (efficacy), we will evaluate the efficacy of CS PTSD Coach in reducing symptoms of PTSD, depression, anxiety, and sleep disturbance when compared with SM PTSD Coach and the waitlist control group. Changes in symptoms are measured using the PCL-5 for PTSD, PHQ-9 for depression, GAD-7 for anxiety, and ISI for sleep disturbance. Exploratory analyses will assess the responsiveness of specific PTSD symptom clusters to the 2 interventions [38]. Within-group changes will be summarized descriptively (mean change and 95% CIs) to aid interpretation.

The primary analysis will compare postintervention (8 weeks) outcomes between intervention groups using ANCOVA, adjusting for baseline outcome values and prespecified covariates (eg, language group). The primary estimand is the between-group difference in change from baseline to 8 weeks.

Separate ANCOVA models will be done to compare between-group changes from baseline to 12-week follow-up (secondary analysis) and from postintervention to 12-week follow-up (exploratory analysis). The sample size and power calculations are applicable to all ANCOVA models.

All analyses will follow an intent-to-treat framework, with missing outcome data addressed through multiple imputation and sensitivity analysis. Exploratory moderation analyses will be conducted by including prespecified interaction terms in ANCOVA models. Exploratory mediation analyses will be conducted using established regression-based approaches.

### Ethical Considerations

Institutional review board (IRB) approval for the study was obtained under protocol IRB-24‐0383 at Florida International University. The informed consent process is conducted online via the REDCap platform by the recruitment team, ensuring secure data collection and participant confidentiality. Participants are assigned unique study IDs, and their data will be deidentified for analysis. Only authorized personnel will have access to identifying information, which will be stored separately and destroyed after the final follow-up assessment. All staff involved in the study completed training in CITI Human Subjects and Good Clinical Practice, meeting ethical standards and data protection.

Safety monitoring is overseen by multiple principal investigators, coinvestigators, and the project coordinator. Biweekly meetings address data safety issues, with monthly reviews involving all investigators. Serious adverse events (SAEs) and adverse events are monitored, with SAEs reported to the university IRB and Centers for Disease Control and Prevention or NIOSH as required. Participants experiencing SAEs or adverse events continue in the study if deemed safe by the IRB and clinical team; otherwise, the assigned intervention is discontinued. Additional safeguards include emergency protocols for participants experiencing psychological distress, including suicidal ideation, and procedures to maintain confidentiality during online sessions. Participants may discontinue their assigned intervention at any time upon request.

## Results

This manuscript describes the protocol for an RCT evaluating CS and SM versions of PTSD Coach among WTC responders living in Florida. The notice of award was received in September 2024, and IRB approval with amendments was granted in January 2025. Recruitment began in February 2025, with data collection beginning with the first participant in March 2025. As of April 2026, 63 participants have been enrolled, and complete outcome data collection is ongoing. Data analysis will commence following completion of follow-up assessments, with results expected to be disseminated in 2027.

Aggregated results will be included in peer-reviewed publications and presentations at conferences. We will also post findings to ClinicalTrials.gov. Findings will be conveyed to NIOSH and other relevant agencies, as required. The aims and details of the projects will also be disseminated to the wider public. The study protocol will be publicly available through trial registration and publication; individual participant data will not be shared due to privacy considerations.

## Discussion

This protocol describes an RCT designed to evaluate the feasibility, acceptability, and efficacy of CS and SM versions of PTSD Coach among WTC GRs residing in Florida. Persistent mental health symptoms, combined with documented barriers to specialty care and geographic dispersion, underscore the need for scalable, accessible interventions tailored to this aging and increasingly mobile population.

The study addresses several critical gaps in the literature. Although PTSD Coach has demonstrated promise among military veteran populations, it has not previously been evaluated with WTC GRs, nor adapted for Spanish-speaking responders. By examining both CS and SM delivery models within a single trial, this study will generate comparative data on the role of human support in enhancing engagement and clinical benefit from digital mental health tools. The inclusion of bilingual delivery further advances equity by addressing linguistic barriers that contribute to unmet mental health needs among Spanish-speaking Hispanic responders.

Key strengths of the study include its randomized controlled design, use of validated outcome measures aligned with the WTCHP monitoring system, and integration of feasibility and acceptability outcomes alongside symptom change. Embedding the intervention within the context of the WTCHP and the National Provider Network enhances clinical relevance and positions the findings to inform real-world implementation should the intervention prove effective.

Several anticipated limitations warrant consideration. Recruitment may be challenged by responder aging, comorbid medical conditions, and variability in comfort with mobile technology. Attrition is a known risk in app-based interventions and may differentially affect study arms. In addition, findings may not generalize to responders living outside Florida or to those without access to smartphones or stable internet connectivity. These factors will be carefully monitored and reported to inform interpretation of study outcomes.

If successful, this trial has the potential to inform scalable mental health service delivery models for WTC responders and other disaster-exposed populations facing similar access barriers. Results will guide future effectiveness and implementation studies and may support broader integration of clinician-supported digital interventions within occupational health and disaster response systems.

## Supplementary material

10.2196/95229 Checklist 1 SPIRIT checklist.

10.2196/95229 Peer Review Report 1 Peer review report from ZOH1 LEE (52) - Disease, Disability and Injury Prevention and Control Special Emphasis Panel, WTC Research Review, Centers for Disease Control and Prevention-National Institute for Occupational Health and Safety.

## Abbreviations

ANCOVA: analysis of covariance
CS: clinician-supported
GAD-7: Generalized Anxiety Disorder-7
GR: general responder
GRDC: General Responder Data Center
IRB: institutional review board
ISI: Insomnia Severity Index
NIOSH: National Institute for Occupational Safety and Health
PCL-5: PTSD Checklist for DSM-5
PHQ-9: Patient Health Questionnaire
PTSD: posttraumatic stress disorder
RCT: randomized controlled trial
SAE: serious adverse event
SM: self-managed
VA: Veterans Affairs
WTC: World Trade Center
WTCHP: World Trade Center Health Program
