# Supplementary material for: A Clinician-Supported Mobile App to Reduce Mental Health Symptoms Among World Trade Center Responders in Florida: Protocol for a Randomized Controlled Trial
Source: JMIR Res Protoc. 2026 Jun 30;15:e95229. doi: 10.2196/95229 (PMC13318202; doi:10.2196/95229)
Supplement: Checklist 1 [file resprot-v15-e95229-s001.docx]

# Multimedia Appendix: SPIRIT 2013 Checklist

**Manuscript**: Clinician-Supported Mobile Application to Reduce Mental Health Symptoms Among World Trade Center Responders in Florida: Protocol for a Randomized Controlled Trial

**Trial registration**: ClinicalTrials.gov (NCT06648928)

## Administrative information

| **Item** | **Checklist item** | **Location in manuscript** |
| --- | --- | --- |
| 1 | Title: descriptive title identifying study design, population, intervention | Title |
| 2a | Trial registration: registry name and identifier | Methods > Study Design |
| 2b | Trial registration dataset (WHO items) | Methods > Study Design (registration ID); other WHO dataset items not fully enumerated |
| 3 | Protocol version | Version 2.0; dated February 16, 2026. |
| 4 | Funding | Funding section (publisher‑formatted) |
| 5a | Roles and responsibilities (contributors, affiliations) | JMIR submission metadata / Title page (publisher‑formatted) |
| 5b | Name/contact for trial sponsor | Florida International University |
| 5c | Role of sponsor/funders | CDC/NIOSH (financial support). The sponsor and funders have no other role (e.g., study design; data collection, management) |
| 5d | Committees/oversight (eg, steering committee, DMC) | Methods > Ethical Considerations |

## Introduction

| **Item** | **Checklist item** | **Location in manuscript** |
| --- | --- | --- |
| 6a | Background and rationale | Introduction > Background and Rationale |
| 6b | Explanation for choice of comparators | Abstract > Methods; Methods > Study Design |
| 7 | Objectives | Abstract > Objective; Introduction > Aims of the Study |
| 8 | Trial design (type, allocation ratio, framework) | Abstract > Methods; Methods > Study Design; Methods > Sample Size Calculation |

## Methods: Participants, interventions, and outcomes

| **Item** | **Checklist item** | **Location in manuscript** |
| --- | --- | --- |
| 9 | Study setting | Methods > Participants; Methods > Recruitment |
| 10 | Eligibility criteria | Methods > Participants |
| 11a | Interventions: detailed description for each group | Methods > Interventions; Methods > Randomization and Groups |
| 11b | Criteria for discontinuing/modifying allocated interventions | Methods > Ethical Considerations |
| 11c | Strategies to improve adherence | Methods > Interventions (fidelity checklists; consultation; independent fidelity assessment) |
| 11d | Relevant concomitant care | Methods > Participants (exclusion: current counseling for PTSD); other concomitant care not explicit |
| 12 | Outcomes (primary/secondary), measurement variables, time points | Abstract > Methods; Methods > Measures |
| 13 | Participant timeline | Figure 1; Methods > Randomization and Groups |
| 14 | Sample size | Methods > Sample Size Calculation |
| 15 | Recruitment | Methods > Recruitment |

## Methods: Assignment of interventions

| **Item** | **Checklist item** | **Location in manuscript** |
| --- | --- | --- |
| 16a | Sequence generation | Methods > Randomization and Groups (random number generator; randomizer.org) |
| 16b | Allocation concealment mechanism | Methods > Randomization and Groups |
| 16c | Implementation (who generates sequence, enrolls, assigns) | Methods > Randomization and Groups |
| 17a | Blinding (who blinded) | Methods > Measures |
| 17b | Blinding procedure/how maintained | Methods > Measures |
| 17c | Circumstances for unblinding | Not applicable; no unblinding procedures planned |

## Methods: Data collection, management, and analysis

| **Item** | **Checklist item** | **Location in manuscript** |
| --- | --- | --- |
| 18a | Data collection methods and assessment schedule | Methods > Measures; Methods > Data Collection and Management |
| 18b | Plans to promote retention and complete follow-up | Methods > Measures |
| 19 | Data management (entry, coding, security, storage) | Methods > Data Collection and Management |
| 20a | Statistical methods for primary and secondary outcomes | Methods > Statistical Analysis Plan |
| 20b | Methods for additional analyses (eg, moderators/mediators) | Methods > Statistical Analysis Plan |
| 20c | Missing data | Methods > Statistical Analysis Plan (multiple imputation; sensitivity analysis) |

## Methods: Monitoring

| **Item** | **Checklist item** | **Location in manuscript** |
| --- | --- | --- |
| 21a | Data monitoring committee (DMC) composition/role | Not applicable; due to the low‑risk nature of the study, no independent data monitoring committee was established. Safety monitoring is conducted by the investigator team and IRB. |
| 21b | Interim analyses and stopping guidelines | Not applicable; no interim analyses or formal stopping guidelines planned. |
| 22 | Harms (AE/SAE collection and reporting) | Methods > Ethical Considerations |
| 23 | Auditing | Not applicable; no independent auditing procedures are planned beyond routine oversight by the IRB and institutional policies. |

## Ethics and dissemination

| **Item** | **Checklist item** | **Location in manuscript** |
| --- | --- | --- |
| 24 | Research ethics approval | Methods > Ethical Considerations (IRB-24-0383) |
| 25 | Protocol amendments | Not stated in manuscript; described here: Substantial protocol amendments are communicated to the IRB and updated in the trial registry prior to implementation. |
| 26a | Consent or assent | Methods > Ethical Considerations (online consent via REDCap) |
| 26b | Additional consent provisions | Not applicable; no additional consent provisions beyond the primary study consent are required. |
| 27 | Confidentiality | Methods > Data Collection and Management; Methods > Ethical Considerations |
| 28 | Declaration of interests | The authors declare no conflicts of interest. |
| 29 | Access to data | Methods > Data Collection and Management (de-identified export for analysis; detailed access statement not explicit) |
| 30 | Ancillary and post-trial care | Methods > Randomization and Groups (wait-list offered CS/SM after follow-up) |
| 31a | Dissemination policy | Results (plans for publications, presentations, posting to ClinicalTrials.gov) |
| 31b | Authorship eligibility guidelines | Not stated in manuscript; Authorship follows established journal authorship criteria. |
| 31c | Plans for sharing protocol/data/materials | Results |

## Appendices

| **Item** | **Checklist item** | **Location in manuscript** |
| --- | --- | --- |
| 32 | Model consent form and related documentation | Consent process described; model consent form not included in the manuscript. |
| 33 | Biological specimens | Not applicable (no specimens) |
