# Supplementary material for: A Clinician-Supported Mobile App to Reduce Mental Health Symptoms Among World Trade Center Responders in Florida: Protocol for a Randomized Controlled Trial
Source: JMIR Res Protoc. 2026 Jun 30;15:e95229. doi: 10.2196/95229 (PMC13318202; doi:10.2196/95229)
Supplement: Peer Review Report 1 [file resprot-v15-e95229-s002.pdf]

SUMMARY STATEMENT

| | | |
|---|---|---|
| PROGRAM CONTACT:<br>JAMES YIIN<br>jcy5@cdc.gov | ( Privileged Communication ) | *Release Date:* 06/20/2024<br>*Revised Date:* |

*Application Number:* 1U01OH012773-01

Principal Investigators (Listed Alphabetically):

LUCCHINI, ROBERTO G
MACGOWAN, MARK J (Contact)

Applicant Organization: FLORIDA INTERNATIONAL UNIVERSITY

*Review Group:* ZOH1 LEE (52)
Disease, Disability and Injury Prevention and Control Special Emphasis Panel
WTC Research Review

*Meeting Date:* 05/28/2024
*Council:* MAY 2024
*Requested Start:* 07/01/2024
*Opportunity Number:* PAR-20-280
*PCC:* WTWTXXXX

*Project Title:* Clinician-Supported Mobile App to Reduce Mental Health Symptoms Among World Trade Center Responders in Florida.
*SRG Action:* Impact Score:36

Human Subjects: 30-Human subjects involved - Certified, no SRG concerns
Animal Subjects: 10-No live vertebrate animals involved for competing appl.
Gender: 1A-Both genders, scientifically acceptable
Minority: 1A-Minorities and non-minorities, scientifically acceptable
Age: 3A-No children included, scientifically acceptable
Clinical Research - not NIH-defined Phase III Trial

| Project Year | Direct Costs Requested | Estimated Total Cost |
|---|---|---|
| 1 | 226,998 | 344,254 |
| 2 | 234,496 | 355,625 |
| TOTAL | 461,494 | 699,879 |

ADMINISTRATIVE BUDGET NOTE: The budget shown is the requested budget and has not been adjusted to reflect any recommendations made by reviewers. If an award is planned, the costs will be calculated by Institute grants management staff based on the recommendations outlined below in the COMMITTEE BUDGET RECOMMENDATIONS section.

**1U01OH012773-01 Macgowan, Mark**
**Clinician-Supported Mobile App to Reduce Mental Health Symptoms Among World Trade Center Responders in Florida**
**SCORE: 36**

**RESUME AND SUMMARY OF DISCUSSION:** This is a new application from an experienced Principal Investigator (PI) with the major goals of evaluating the use of an existing and readily available mobile app (PTSD Coach) to provide posttraumatic stress disorder (PTSD) treatment for a primarily Latinx sample of World Trade Center (WTC) general responders now living in Florida. This randomized clinical trial will have three arms: 1.) PTSD Coach app + clinician support (CS PTSD Coach), 2.) PTSD Coach app alone (self-managed, or SM PTSD Coach), and 3.) a control arm of wait listed individuals. The study will examine the feasibility, acceptability, and efficacy of the two interventions on the primary outcome (reduction of PTSD symptoms) and three other secondary outcomes (depression, anxiety, and sleep disturbance). CS PTSD Coach has previously been validated by and disseminated within the Veterans Health Administration for use in English speaking military veterans. The team proposes to develop and deliver a Spanish language version of the app for greater accessibility in underserved populations of WTC general responders in South Florida. This is a highly significant application due to its focus on bringing remote access mental healthcare to general responders outside of the New York City (NYC) area and those who identify as Latinx. It is innovative in that it will translate a mobile app largely used in military veterans to a civilian population with a mass casualty form of trauma. The PI is outstanding and has expertise in mass casualty trauma and remote mental health administration as well as a history of externally funded intervention research. In addition to the PI, the strengths of the application include: there is a need to reach general responders who have moved away from NYC and to provide remote mental healthcare resources to both English and Spanish speakers; the study builds on a strong foundation of prior work and has broad implications; it will determine the differences in use and engagement between SM PTSD Coach and CS PTSD Coach in this population, which has important differences from military veterans; the measures to be used to determine presence and severity of symptoms of PTSD, depression, alcohol mis/use, anxiety, and sleep disturbances are all sound and consistent with what is commonly used in the field; the inclusion of an onsite clinician to supervise CS PTSD Coach is a strength of the design and important for ensuring fidelity; there are strong preliminary data regarding interest in participating in studies and documentation of PTSD and other mental health issues among this at-risk population; the methods and statistical analysis plan are strong; and the environment is excellent. The weaknesses include: there is satisfactory existing evidence that the efficacy of CS PTSD Coach is greater than SM PTSD Coach; the three aims are interdependent; the proposed sample sizes for each intervention group are unlikely to be sufficient; and the number of general responders living in South Florida who have consented to research activities is relatively small and may not be representative of all general responders in this area. There are no human subjects issues and inclusion is adequate with respect to gender, minority status, and age. There are no budget issues. The application's strengths outweigh its weaknesses.

**DESCRIPTION (provided by applicant):** The World Trade Center (WTC) General Responders (GRs) carry a significant mental health (MH) burden from the exposure to intense psychological trauma after the WTC collapse. PTSD and depression are two of the top ten certifiable mental health conditions that the 9/11 GRs are burdened with. Members of the WTC Health Program (WTCHP), including GRs, FDNY and Survivors, have increasingly moved to Florida representing close to six percent of the cohort. They are away from the NY Metropolitan Area WTCHP, creating a need for localized, accessible care. Additionally, preliminary findings from our ongoing pilot study on the WTCHP GRs residing in Florida, indicate a large majority of the participants identify as Latinx, creating a need to provide accessible services in the language of their choice, addressing health equity. The findings also

indicate that the majority prefer individual care and prefer, or are open to, remotely delivered services. While many studies have identified the MH burden that the responders carry, there have been no controlled studies of psychosocial interventions to reduce PTSD, depression, anxiety, and sleep symptoms among multiethnic GRs. There is a critical need for developing or testing interventions appropriate for the GRs. Preliminary evidence suggests that remotely delivered and clinician-supported interventions can reduce adverse MH symptoms as part of low-cost approaches with scalability potential in adults with significant MH conditions. The goal of this proposed U01 2-year study from Principal Investigators at a Hispanic Serving Institution (HSI), is to assess the feasibility, acceptability, and efficacy of Clinician-Supported PTSD Coach, originally developed for English speaking military veterans but never tested with responders, for reducing symptoms of PTSD, depression, anxiety, and sleep disturbance among English and Spanish speaking WTC GRs. PTSD Coach is a mobile app available at no cost for Apple and Android mobile devices. PTSD Coach is a self-managed (SM) application but given the need for more personalized, synchronous, and attentive services, the clinician-supported (CS) PTSD Coach intervention was developed. Studies of CS PTSD Coach with English-speaking veterans reported reductions in PTSD symptoms. Collectively, these preliminary data suggest CS PTSD Coach is a promising intervention for the GRs in Florida. Findings from this study with a known cohort in Florida will inform the deployment of an expanded trial nationally on CS PTSD Coach with WTC GRs.

**PUBLIC HEALTH RELEVANCE:** World Trade Center (WTC) general responders (GR) carry a significant public mental health (MH) burden related to their work in the WTC collapse. Many have increasingly moved to Florida, representing a substantial and ethnically diverse number of GRs who are away from their NY-based WTC support system, creating a need for personalized, accessible MH care. Responsive to the need to (a) develop efficacious MH treatments for the WTC responders (Zadroga Act, Title 42 USC §300mm–51) and (b) advance health equity, this research application from MPIs from a Hispanic-Serving Institution proposes to assess the feasibility, acceptability, and efficacy of Clinician-Supported (CS) PTSD Coach, originally developed for English speaking military veterans but never tested with WTC responders, for reducing symptoms of PTSD, depression, anxiety, and sleep disturbance among English and Spanish speaking WTC GRs. If successful with the Florida cohort, CS PTSD Coach will be a readily deployable intervention to reduce PTSD, depression, and related symptoms among the dispersed English- and Spanish-speaking WTC GRs.

**CRITIQUE 1**

Significance: 1
Investigator(s): 1
Innovation: 2
Approach: 2
Environment: 1

**Overall Impact:** This is a new application from an outstanding Principal Investigator (PI) and investigative team with the major goals of evaluating the use of an existing and readily available mobile app (PTSD Coach) to provide posttraumatic stress disorder (PTSD) treatment for a primarily Latinx sample of World Trade Center (WTC) general responders now living in Florida. This is a highly significant area because of its focus on general responders who have left the New York City (NYC) area and those who identify as Latinx. It is innovative for its focus on an underserved community that has not received substantial attention in the WTC cohort or the broader mental health literature. The major strengths of the application are the use an established intervention, a prior record of feasibility

regarding recruitment, the involvement of the mobile application developers, and the focus on the Latinx provider community outside of NYC. Its weaknesses are an ambitious timeline and the focus on large effects as the outcome. By and large, these weaknesses are addressable. The application's strengths greatly outweigh its weaknesses.

**Strengths**

- Major: The study builds on a strong foundation of prior work and has broad implications.

**Weaknesses**

- Minor: There are concerns with the size of the sample to adequately detect the range of effects that are likely to be observed.

**1. Significance:**

**Strengths**

- Major: Determining the differences in engagement between clinician supported and self-directed use of the intervention is highly significant.
- Major: Focusing on use and engagement patterns is outstanding and important.
- Major: Examining engagement across languages is very important.

**Weaknesses**

- Minor: The extant evidence points to the relatively improved efficacy of Clinician Supported (CS) PTSD Coach over Self-Managed (SM) PTSD Coach. It is unclear why SM PTSD Coach should continue to be evaluated.

**2. Investigator(s):**

**Strengths**

- Major: The investigative team is outstanding.

**Weaknesses**

- None are noted.

**3. Innovation:**

**Strengths**

- Moderate: There is a need to evaluate WTC responders who have left the NYC area.
- Moderate: There is a need for racial/ethnic directed work within the WTC cohort.

**Weaknesses**

- Minor: It is unclear if the SM PTSD Coach version is worthwhile to incorporate into the current study.

**4. Approach:**

**Strengths**

- Major: The use of an established and well studied mobile intervention is a significant strength.
- Major: The availability of PTSD Coach in Spanish is a significant strength.

- Moderate: Translation to Spanish is done appropriately.
- Minor: The examination of changes in the clusters of PTSD in addition to the overall diagnosis is a relative strength.

**Weaknesses**

- Moderate: The proposed effect size under investigation is quite large. There is a concern that the sample size is insufficient to detect the likely smaller effects that will be observed and thus incorrect conclusions about the efficacy of PTSD Coach could be drawn.
- Minor: In Aim 1, use of the app would benefit from benchmarks for success. It is unclear what would be considered successful use of the app.
- Minor: The sample is likely under powered to detect meaningful differences in outcomes for different moderators. This is viewed as a minor weakness as this aim is largely exploratory.

**5. Environment:**

**Strengths**

- The environment is outstanding.

**Weaknesses**

- None are noted.

**Study Timeline:**

**Strengths**

- None are noted.

**Weaknesses**

- The timeline is ambitious given the two-year period.

**Protections for Human Subjects**

Acceptable Risks and/or Adequate Protections

Data and Safety Monitoring Plan (Applicable for Clinical Trials Only):

Acceptable

**Inclusion Plans (Applicable Only for Human Subjects research and not IRB Exemption #4.)**

- Sex/Gender: Distribution justified scientifically
- Race/Ethnicity: Distribution justified scientifically
- For NIH-Defined Phase III trials, Plans for valid design and analysis: Not Applicable
- Inclusion/Exclusion Based on Age: Distribution justified scientifically

**Vertebrate Animals**

Not Applicable (No Vertebrate Animals)

**Biohazards**
Not Applicable (No Biohazards)

**Limitations of Currently Available Data**
Acceptable

**Project Dissemination Plan**
Acceptable

**Project Evaluation Plan**
Acceptable

**Select Agents**
Not Applicable (No Select Agents)

**Resource Sharing Plans (including a Data Management Plan)**
Acceptable

**Authentication of Key Biological and/or Chemical Resources**
Not Applicable

**Budget and Period of Support**
Recommend as Requested

**CRITIQUE 2**

Significance: 4
Investigator(s): 3
Innovation: 4
Approach: 4
Environment: 1

**Overall Impact:** This is a new application from an experienced Principal Investigator (PI) with the major goals of assessing the feasibility, acceptability, and efficacy of a clinician supported version of the existing PTSD Coach mobile app that has been validated by and disseminated within the Veterans Health Administration (VA). Additionally, the team proposes to develop and deliver a Spanish-language version of the Clinician Supported (CS) PTSD Coach app for greater accessibility in underserved populations in South Florida who were general responders to the 9/11 attacks on the World Trade Center (WTC). They will leverage the team's ongoing collaboration with the WTC Health Program (WTCHP). This is a significant area of study as it addresses legislation focused on meeting the needs of this traumatized population with expanded access to remote mental health care. It is innovative in

that it will translate a mobile app largely used in veterans to a civilian population with a mass casualty form of trauma. The PI is a professor and associate dean in the Robert Stempel College of Public Health and Social Work at Florida International University (FIU) and has experience with this type of mass casualty trauma and with trauma treatment methods. The major strengths of the application are its potential impact on providing wider access to trauma-focused treatment remotely via a mobile app, its inclusion of comparisons with the existing self-managed version of PTSD Coach and wait list control, and the outcome measures to be examined. Its weaknesses are somewhat limited recruitment strategies, a narrowly focused target population of interest, and the inherent problems that can arise from mobile app usage including participant compliance, boredom, and apathy. There are no human subjects issues and inclusion is adequate with respect to gender, minority group status, and children. By and large, these weaknesses are moderate and addressable, and the application's strengths somewhat outweigh its weaknesses.

**1. Significance:**

**Strengths**

- Major: The primary area of significance for the current proposal is the potential ability to reach WTC general responders who still exhibit clinically relevant PTSD symptoms (full diagnosis or sub-threshold) through a smart phone application that includes clinician support, an enhanced version of a series of “Coach” apps developed largely in cooperation with the National Center for PTSD with veteran study samples. The group of traumatized individuals are those who left the New York City area and relocated to South Florida. This work is in line with legislation focused on the treatment of WTC responders in areas of uncertainty.
- Moderate: Developing an app for primarily Spanish speakers is significant given the potential implications for expanding access to mental health care in underserved areas and populations.
- Moderate: The examination of the Spanish version of the CS PTSD Coach may provide insight into other efforts to translate the application for more widespread domestic and international use.

**Weaknesses**

- Moderate: The authors are targeting a relatively small group of general responders (1,450 who have consented to research activities out of approximately 5,500-7,000+ who now live in South Florida). There are some concerns about generalizability of the data beyond this cohort.

**2. Investigator(s):**

**Strengths**

- Major: The PI is a professor and associate dean in the School of Public Health at FIU with a history of externally funded intervention research including development, assessment, and testing phases. Additionally, he has expertise in mass casualty trauma (including recent tragedies in South Florida) and remote mental health administration. Co-Investigator Lucchini is a full professor at FIU in the School of Public Health and Social Work and the former Director of the World Trade Center Health Program (WTCHP) and has been well funded in this area of work. Dr. Kuhn brings significant experience and expertise in the adoption and use of technology in mental health care with different levels of provider involvement across a variety of modalities. Dr. Possemato provides considerable experience with the application of study, CS PTSD Coach, having been centrally involved in its development. Complementary staff and investigator support in biostatistics, mental health assessment, and data management are readily available, and this is a strength of the team.

**Weaknesses**

- Minor: It is not immediately apparent how much experience the proposed FIU team (save Dr. Lucchini) has with long-term survivors of temporally remote mass casualty events such as the 9/11 attacks on the WTC, although experience with the more recent Surfside tower collapse is noted.

**3. Innovation:**

**Strengths**

- Moderate: The use of a device-borne application to deliver mental health services to a previously traumatized population who are both physically and temporally removed from their traumatic event(s) is innovative as the most compelling data has come largely from studies among veterans. Expanded civilian usage and availability is somewhat innovative, as is the proposed use among Spanish speaking/Latinx WTC responders who have relocated to Florida.
- Moderate: Direct comparison between the SM PTSD Coach and the CS PTSD Coach in this population with this type of trauma is innovative and forward-thinking.

**Weaknesses**

- Moderate: The use of mobile apps in lieu of in-person treatment has been explored for some time and the proposed CS PTSD Coach has met some of the early markers for use elsewhere.

**4. Approach:**

**Strengths**

- Major: The rationale for using PTSD Coach, both self-managed and clinician supported, is strong and based on prior empirical and clinical use of the application.
- Moderate: The measures to be used to determine presence and severity of symptoms of PTSD, depression, alcohol mis/use, anxiety, and sleep disturbances are all sound and consistent with what is commonly used in the field.
- Moderate: The inclusion of an onsite clinician to supervise the clinician supported version of PTSD Coach is a strength of the design and important for ensuring fidelity.
- Moderate: The power analyses and proposed statistical analyses are sound and supported by the inclusion of a biostatistician on the study team.

**Weaknesses**

- Moderate: Sample sizes for the intervention arms may not be sufficient at 40 per group.
- Moderate: The aims appear to be interdependent.
- Minor: Recruitment efforts appear to be based on those who have previously consented to participate in this group’s research (n = 1,450) with some new advertising and de novo recruitment from this subpopulation living in South Florida. This heavy reliance on the consented group may hinder recruitment.

**5. Environment:**

**Strengths**

- Major: The PI and primary co-investigators work within a relatively new (2014) combined complex that affords office, laboratory, conference, and classroom space to support the planned research activities. This space is within the wide-ranging resources of the Stempel College of Public Health and Social Work, the School of Social Work, the Department of Environmental Health Sciences, and the Center for Statistical Consulting and Collaboration. The FIU environment is excellent.
- Major: Sub-award site VA Palo Alto provides an excellent scientific environment and support for the collaborative study team with additional support from neighboring Stanford University. Additionally, VA Palo Alto benefits from a close association with the National Center for PTSD (NCPTSD) and the NCPTSD Mobile Mental Health Program.
- Moderate: Sub-award site VA Center for Integrated Healthcare provides valuable support and expertise in the integration of mental health services into primary care through the development and implementation of brief interventions.

**Weaknesses**

- None are noted.

**Study Timeline:**

**Strengths**

- The number of participants is feasible for the proposed timeline provided.

**Weaknesses**

- The initial 1-3 months of the proposed study timeline has at least six preparatory sub-tasks. This may not be sufficient time to accomplish these.

**Protections for Human Subjects**

Acceptable Risks and/or Adequate Protections

- There were no apparent concerns regarding human subjects protection or risk. Onsite clinical supervisor and clinical members of the team are a strength.

Data and Safety Monitoring Plan (Applicable for Clinical Trials Only):

Acceptable

- There were no apparent concerns regarding the DSMP.

**Inclusion Plans (Applicable Only for Human Subjects research and not IRB Exemption #4.)**

- Sex/Gender: Distribution justified scientifically
- Race/Ethnicity: Distribution justified scientifically
- For NIH-Defined Phase III trials, Plans for valid design and analysis:
- Inclusion/Exclusion Based on Age: Distribution justified scientifically
  - The inclusion of women and minorities is acceptable. Most notably, Latinx members of the population are a sample of interest to the investigators and are justifiably included with anticipated higher numbers compared to other groups. This is focused on individuals who likely retired to Florida from New York City and will skew older.

**Vertebrate Animals**
Not Applicable (No Vertebrate Animals)

**Biohazards**
Not Applicable (No Biohazards)

**Dual Use Research of Concern**
Not Applicable

**Limitations of Currently Available Data**
Acceptable

- There are no significant concerns.

**Project Dissemination Plan**
Acceptable

- The Project Dissemination Plan is appropriate for the work proposed and includes government, scientific, media, and public forums as well as presentation and publication in the peer-reviewed literature.

**Project Evaluation Plan**
Unacceptable

- Very little detail is presented in the Evaluation Plan included in the application.

**Select Agents**
Not Applicable (No Select Agents)

**Resource Sharing Plans (including a Data Management Plan)**
Acceptable

- Measures are in place and appropriate.

**Authentication of Key Biological and/or Chemical Resources**
Not Applicable

**Budget and Period of Support**
Recommend as Requested

## CRITIQUE 3

**Overall Impact:** This new two-year U01 application proposes to examine the feasibility (e.g., objective data assessing mobile app engagement), acceptability (e.g., participant satisfaction), and efficacy of two active intervention conditions [i.e., clinician supported sessions + a PTSD Coach app or self-management with the PTSD Coach app (app alone)] relative to a wait list control group in a randomized trial on the primary outcome (reduction of PTSD symptoms) and three other secondary outcomes (depression, anxiety, and sleep disturbance) among WTCHP members living in Florida. The application has a considerable number of major strengths: a well-argued and strong empirical rationale for examining whether the PTSD Coach app, developed by one of the co-investigators for English-speaking military veterans, is relevant and impactful for WTCHP general responders in Florida, including older adults, Latinx ethnicity, as well as a subgroup of Spanish-speaking responders; excellent investigative team who integrate expertise in the WTC population, mental health, and app development; strong preliminary survey data regarding the interest of WTCHP general responders to participate in studies and documentation of PTSD and other mental health issues among this at-risk population; many extremely strong methodological aspects for the conduct of the proposed studies including details about recruitment pools and rates, a honed set of validated assessments with explicit mention of clinical relevance/specific cut points, ensuring blinded assessment staff, and details about training interventionists and intervention fidelity. There were two moderate-to-major weaknesses. First, the three aims are interdependent; if the WTCHP general responders do not engage with the app as assessed in Aim 1, the efficacy in Aim 3 cannot be assessed as the study design relies on the same 120 study participants. Given the three-year possible timing for the U01, it is unclear why Aims 1 and 2 are not first conducted using a smaller number of study participants (consistent with feasibility and acceptability study sizes) before recruiting the set of 120 study participants for Aim 3. Second, the proposed sample sizes for the trial and three intervention conditions, despite the numbers used in the power calculation, seem unlikely to be sufficient (only n=40 in each intervention condition with an ambitious attrition rate of only 10%). The preliminary data provided about the large, multisite clinical trial in a related population (n=234) only showed trends for similar outcome measures in a two-arm trial. There were also an enormous number of proposed sub-analyses with no discussion of any corrections for the number of tests. There were no human subjects issues (e.g., Research Protection of Human Subjects section was detailed and thorough), and inclusion was adequate with respect to gender, minority group status and children. The weaknesses, although moderate to major, are addressable, and overall, enthusiasm is high for this new U01 application.

**Study Timeline:**

**Strengths**

- The timeline cleanly laid out not only basic study tasks but also estimated study participant accrual by month.

**Weaknesses**

- None are noted.

**Protections for Human Subjects**

Acceptable Risks and/or Adequate Protections

- This was appropriately detailed and consistent with the Research Plan. For instance, there was a detailed section on managing risk of suicide during study recruitment (i.e., how to exclude

potential participants at risk of suicide which also provided explicit follow-up procedures for assessors with at-risk potential participants during screening).

Data and Safety Monitoring Plan (Applicable for Clinical Trials Only):

Acceptable

- A thorough and detailed DSMP was provided.

**Inclusion Plans (Applicable Only for Human Subjects research and not IRB Exemption #4.)**

- Sex/Gender: Distribution justified scientifically
- Race/Ethnicity: Distribution justified scientifically
- For NIH-Defined Phase III trials, Plans for valid design and analysis: Scientifically Acceptable
- Inclusion/Exclusion Based on Age: Distribution justified scientifically
  - Although the race/ethnicity distribution was justified scientifically, i.e., to reflect the diversity of the WTCHP members currently living in Florida, no specific recruitment strategies are described for the recruitment of ethnic minority adults.

**Vertebrate Animals**

Not Applicable (No Vertebrate Animals)

**Biohazards**

Not Applicable (No Biohazards)

**Dual Use Research of Concern**

Not Applicable

**Limitations of Currently Available Data**

Not applicable (No Currently Available Data)

**Project Dissemination Plan**

Acceptable

- A detailed and thorough plan was provided including a list and descriptions of five different targeted stakeholders and six dissemination tools.

**Project Evaluation Plan**

Acceptable

- This plan included how the team would meet and interact given their different roles, but did not include specific evaluation measures.

**Select Agents**

Not Applicable (No Select Agents)

**Resource Sharing Plans (including a Data Management Plan)**

Acceptable

- Procedures were summarized for the sharing of the new data generated by this application and the WTCHP data sets.

**Authentication of Key Biological and/or Chemical Resources**

Not Applicable

**Budget and Period of Support**

Recommend as Requested

**THE FOLLOWING SECTIONS WERE PREPARED BY THE SCIENTIFIC REVIEW OFFICER TO SUMMARIZE THE OUTCOME OF DISCUSSIONS OF THE REVIEW COMMITTEE, OR REVIEWERS' WRITTEN CRITIQUES, ON THE FOLLOWING ISSUES:**

**PROTECTION OF HUMAN SUBJECTS: ACCEPTABLE**

**INCLUSION OF WOMEN PLAN: ACCEPTABLE**

**INCLUSION OF MINORITIES PLAN: ACCEPTABLE**

**INCLUSION ACROSS THE LIFESPAN: ACCEPTABLE**

**COMMITTEE BUDGET RECOMMENDATIONS: The budget was recommended as requested.**
